# Supplementary material for: A Likelihood Approach for Real-Time Calibration of Stochastic Compartmental Epidemic Models
Source: PLoS Comput Biol. 2017 Jan 17;13(1):e1005257. doi: 10.1371/journal.pcbi.1005257 (PMC5240920; doi:10.1371/journal.pcbi.1005257)
Supplement: S1 File — (TAR.GZ) [file pcbi.1005257.s014.tar.gz › HSPH_Online-SI-Revision/Additions/EnKF08/n100-extreme_noisy/n100-extreme_noisy_EnKF_table-4.pdf]

1	R0	{2.76052, 16.3913}	{1.92205, 8.52085}	{{0.351284, 2.55485}, {3., 13.048}}	{38.7019, 48.016}	inf	10 419.	4175.81	{252.71, 2695.36}	91.3082	0.903691	newly	5869.58	3625.7	{147., 4661.}	98.6442	0.834253	Reff	2.28524	0.377556	{0.0729913, 0.642473}	83.6926	ar	92 090.	186 303.	{174 239., 204 475.}	102.305
2	R0	{2.18202, 15.1734}	{2.68388, 3.97048}	{{2.16627, 1.74649}, {3., 6.13496}}	{23.1878, 73.8326}	inf	10 970.	7779.69	{677.355, 8775.04}	102.839	0.633179	newly	5914.	13 977.4	{999., 12 556.}	219.796	0.433534	Reff	1.6841	0.579155	{0.106299, 0.780777}	70.663	ar	84 264.	201 152.	{179 717., 234 555.}	138.716
3	R0	{2.57891, 12.5971}	{2.52805, 6.65755}	{{1.42222, 3.26913}, {3., 9.67824}}	{20.5864, 47.1501}	inf	8525.	2888.74	{129.797, 9011.86}	82.8576	0.86104	newly	6571.	4000.55	{78., 7234.}	102.462	0.865846	Reff	2.202	0.564676	{0.108381, 1.20889}	74.6202	ar	90 302.	185 552.	{169 621., 237 662.}	105.479
4	R0	{2.5856, 9.28508}	{2.33416, 4.15778}	{{1.77271, 0.911885}, {3., 12.9812}}	{24.1491, 68.8979}	inf	2564.	22 283.5	{4228.55, 58 281.5}	772.89	0.805593	newly	3492.	27 057.4	{4271., 71 264.}	684.453	0.754983	Reff	2.47933	0.523279	{0.188311, 1.0316}	78.8944	ar	90 312.	157 177.	{94 341., 250 326.}	81.395
5	R0	{2.4406, 12.1341}	{2.46668, 4.60195}	{{1.99232, 1.57083}, {3., 7.24475}}	{21.8521, 62.0742}	inf	4472.	1601.62	{852.576, 2070.7}	74.5861	0.582605	newly	3644.	2606.06	{724., 4284.}	86.8463	0.508983	Reff	2.2517	0.542557	{0.169494, 1.62159}	75.9045	ar	88 597.	190 302.	{180 424., 244 830.}	118.963
6	R0	{2.72155, 15.4094}	{2.10839, 9.0161}	{{0.309638, 2.86529}, {3., 12.7854}}	{34.1936, 41.4896}	inf	8392.	3930.	{124.289, 3319.17}	98.498	1.01363	newly	5118.	2736.01	{65., 4788.}	96.6075	0.98394	Reff	2.34317	0.375548	{0.0652878, 0.649956}	83.9727	ar	91 682.	189 234.	{180 882., 196 536.}	106.402
7	R0	{2.63781, 14.3935}	{2.25429, 5.87231}	{{0.140455, 3.46147}, {3., 12.5564}}	{31.0162, 59.2017}	inf	9079.	10 021.9	{243.571, 9698.26}	123.039	0.677755	newly	5875.	8790.99	{356., 20 482.}	131.937	0.553992	Reff	2.22919	0.585905	{0.0416845, 0.747474}	73.7166	ar	91 192.	194 674.	{169 548., 253 132.}	113.478
8	R0	{2.82391, 16.104}	{2.21988, 7.81603}	{{0.36396, 2.80774}, {3., 12.8152}}	{28.748, 51.4653}	inf	8948.	3276.81	{383.149, 2618.13}	77.6677	0.728838	newly	5170.	2865.6	{225., 3742.}	74.9037	0.633404	Reff	2.41478	0.443414	{0.047712, 0.608187}	81.6375	ar	92 664.	183 573.	{176 286., 183 516.}	98.1058
9	R0	{2.80645, 14.6457}	{2.04587, 8.22445}	{{0.37284, 2.94234}, {3., 13.0226}}	{33.7217, 43.8439}	inf	5809.	3989.34	{2757.54, 4574.43}	65.0018	0.365246	newly	4055.	3680.97	{1162., 11 786.}	80.9186	0.41088	Reff	2.54301	0.415596	{0.071266, 0.630281}	83.6652	ar	92 567.	183 349.	{174 854., 218 522.}	98.0721
10	R0	{1.86213, 5.4271}	{2.22327, 4.01613}	{{1.04839, 2.64718}, {3., 4.92073}}	{48.5087, 25.9986}	inf	1977.	15 377.7	{7201.19, 36 480.2}	682.104	0.771104	newly	4393.	16 067.4	{6656., 42 695.}	271.283	0.44219	Reff	1.77612	0.313646	{0.0458242, 0.579037}	82.3409	ar	75 362.	163 620.	{115 437., 231 406.}	118.982
11	R0	{2.27349, 14.6338}	{2.57236, 5.8446}	{{1.60894, 3.48152}, {3., 11.7732}}	{30.8911, 60.061}	inf	10 491.	9527.69	{128.979, 4494.17}	113.417	0.737028	newly	6044.	8869.63	{153., 30 994.}	120.749	0.5456	Reff	1.80677	0.682654	{0.030673, 0.89118}	65.183	ar	85 805.	195 995.	{168 970., 288 541.}	128.419
12	R0	{2.77261, 14.1894}	{2.42014, 13.2294}	{{1.48736, 4.9773}, {3., 18.9945}}	{19.7656, 40.9787}	inf	6492.	4396.99	{433.191, 3424.71}	92.8908	0.661007	newly	4508.	5871.31	{149., 9687.}	155.409	0.825357	Reff	2.48165	0.8078	{0.173925, 2.16046}	67.4491	ar	92 508.	193 055.	{169 351., 256 108.}	108.691
13	R0	{2.65402, 19.3935}	{2.34023, 7.23803}	{{1.00333, 0.825032}, {3., 18.3766}}	{27.727, 62.6781}	inf	11 250.	7428.65	{1582.54, 6717.83}	65.3973	0.439656	newly	4926.	10 094.6	{551., 14 666.}	165.288	0.518983	Reff	2.14402	0.474711	{0.0686637, 0.673553}	78.4353	ar	91 184.	203 238.	{182 979., 241 251.}	122.888
14	R0	{1.75297, 4.45354}	{2.58992, 4.83568}	{{2.00491, 1.26934}, {3., 7.51965}}	{51.593, 58.82}	inf	699.	1892.96	{373.394, 2183.97}	187.348	0.319671	newly	1855.	3134.24	{436., 4342.}	159.847	0.480129	Reff	1.72278	0.451346	{0.128663, 0.976039}	74.6735	ar	71 263.	177 922.	{166 611., 196 238.}	149.67
15	R0	{1.99928, 7.1501}	{2.47412, 5.4051}	{{1.02822, 1.20609}, {3., 7.48225}}	{34.7787, 29.5399}	inf	3389.	2658.65	{597.243, 3074.61}	85.4761	0.42781	newly	4922.	3827.72	{675., 5017.}	105.806	0.617826	Reff	1.85853	0.483016	{0.172308, 1.00395}	75.0672	ar	79 602.	187 041.	{175 196., 205 924.}	135.911
16	R0	{2.67398, 10.3134}	{2.60648, 4.99191}	{{2.01434, 1.14511}, {3., 7.4538}}	{17.8867, 51.5979}	inf	4133.	2183.93	{697.489, 4001.71}	82.5076	0.567113	newly	4823.	2873.3	{662., 3837.}	96.4255	0.65827	Reff	2.49563	0.415344	{0.160684, 0.558648}	83.3571	ar	91 289.	166 589.	{159 126., 189 663.}	85.5468
17	R0	{2.44634, 16.3226}	{2.27806, 5.40737}	{{0.598787, 0.809372}, {3., 18.3822}}	{32.3665, 71.9241}	inf	10 530.	11 092.9	{1286.08, 7210.82}	78.3353	0.331257	newly	5419.	19 320.2	{459., 73 069.}	289.23	0.510681	Reff	1.9738	0.586737	{0.114273, 0.78099}	73.2285	ar	88 710.	191 730.	{168 192., 264 049.}	116.131
18	R0	{2.02168, 10.1676}	{2.14299, 5.99589}	{{0.1321, 3.46246}, {3., 11.9571}}	{46.2625, 49.8295}	inf	4597.	9506.03	{149.404, 14 787.3}	135.349	0.360293	newly	4153.	8186.55	{134., 11 852.}	142.318	0.429208	Reff	1.82331	0.461408	{0.0307112, 0.676653}	74.7137	ar	80 299.	193 001.	{172 287., 203 772.}	140.353
19	R0	{2.89969, 15.009}	{2.10983, 8.44389}	{{0.386119, 2.92455}, {3., 13.0251}}	{30.9063, 43.7412}	inf	6390.	5437.49	{140.039, 6838.48}	91.5684	0.567542	newly	4254.	4352.31	{65., 18 989.}	96.2019	0.576354	Reff	2.60468	0.388861	{0.0272434, 0.617808}	85.0707	ar	93 349.	193 397.	{180 698., 256 312.}	107.176
20	R0	{2.68933, 7.34928}	{2.54785, 5.30382}	{{1.81415, 1.25809}, {3., 12.6317}}	{18.4301, 68.4522}	inf	478.	24 960.8	{4403.52, 59 176.7}	5123.37	1.57413	newly	1059.	27 704.6	{4470., 73 075.}	2517.78	1.24145	Reff	2.66728	0.622132	{0.16473, 1.2674}	76.6754	ar	91 574.	151 467.	{79 935., 252 737.}	71.4256
21	R0	{2.15352, 9.28074}	{2.42173, 4.70236}	{{1.04237, 1.56075}, {3., 7.25143}}	{28.5831, 49.3321}	inf	3961.	2023.99	{800.949, 2818.3}	80.6945	0.558275	newly	4282.	3245.58	{748., 4631.}	101.316	0.620907	Reff	1.99267	0.476751	{0.154996, 1.058}	76.0984	ar	83 845.	187 990.	{172 388., 218 191.}	124.212
22	R0	{2.21953, 9.04387}	{1.99255, 3.66002}	{{0.0262468, 0.906804}, {3., 5.73733}}	{48.9065, 60.7709}	inf	3707.	6225.89	{729.888, 19 553.}	107.91	0.337728	newly	4322.	6895.45	{535., 17 870.}	112.342	0.404896	Reff	2.06581	0.268304	{0.0041346, 0.447239}	87.0121	ar	84 856.	160 609.	{58 443., 222 301.}	99.7415
23	R0	{2.21987, 14.5347}	{2.73191, 4.01956}	{{2.2313, 1.25876}, {3., 6.34132}}	{23.066, 72.345}	inf	10 313.	3455.77	{754.132, 3627.81}	82.2618	0.677862	newly	5905.	6475.37	{1220., 5920.}	110.341	0.40983	Reff	1.76493	0.584557	{0.299575, 0.753509}	68.4316	ar	85 067.	190 093.	{178 711., 207 116.}	123.463
24	R0	{2.84268, 17.9659}	{2.78079, 4.45098}	{{2.35171, 1.87731}, {3., 6.13441}}	{9.59285, 75.2254}	inf	10 277.	4938.6	{2240.38, 3669.}	84.2652	0.607552	newly	5220.	6941.05	{2124., 6380.}	118.219	0.367589	Reff	2.35894	0.449216	{0.208214, 0.708798}	80.9569	ar	92 836.	184 446.	{171 198., 193 669.}	98.6791
25	R0	{2.52706, 10.3679}	{2.51594, 11.8943}	{{1.40773, 4.86063}, {3., 17.7211}}	{20.6504, 48.8143}	inf	4409.	3334.77	{895.011, 2958.06}	76.0681	0.424679	newly	4655.	3936.44	{380., 3305.}	120.951	0.715412	Reff	2.33579	0.644955	{0.251093, 2.12383}	72.3881	ar	89 623.	190 112.	{175 058., 246 470.}	112.124
26	R0	{2.62835, 19.1088}	{2.36499, 5.67744}	{{0.891255, 0.838177}, {3., 18.3521}}	{27.5538, 70.2889}	inf	12 549.	5309.97	{1013.94, 5643.73}	67.9611	0.525132	newly	5505.	9979.09	{873., 13 149.}	138.499	0.443846	Reff	2.05547	0.523207	{0.153395, 0.888713}	75.6165	ar	90 908.	188 785.	{173 335., 233 888.}	107.666
27	R0	{2.76831, 19.4155}	{2.13059, 6.29462}	{{0.470653, 3.47926}, {3., 12.3035}}	{30.9038, 67.5794}	inf	16 382.	11 050.8	{723.112, 35 268.9}	113.681	0.864551	newly	6900.	7734.73	{747., 39 483.}	129.728	0.604103	Reff	1.96727	0.459679	{0.100972, 0.75764}	76.6337	ar	92 175.	203 482.	{179 409., 305 059.}	120.756
28	R0	{2.96609, 16.8903}	{2.6047, 5.02563}	{{1.99541, 1.41701}, {3., 7.19581}}	{13.6245, 70.2455}	inf	11 248.	1553.19	{424.362, 2116.07}	86.7342	1.00153	newly	6075.	2498.8	{453., 4357.}	88.3756	0.749802	Reff	2.44352	0.520917	{0.148942, 1.06523}	78.6817	ar	94 024.	185 210.	{173 267., 222 704.}	96.9819
29	R0	{2.17046, 14.39}	{2.68055, 4.08028}	{{2.18031, 1.13601}, {3., 6.25129}}	{23.5013, 71.645}	inf	8850.	4611.55	{798.511, 7298.43}	85.3559	0.616859	newly	5160.	10 352.8	{1135., 6329.}	198.539	0.470535	Reff	1.78813	0.481776	{0.0909059, 0.638036}	75.8075	ar	84 001.	199 385.	{180 836., 235 200.}	137.361
30	R0	{2.88772, 12.4647}	{2.38268, 5.79634}	{{1.76214, 1.16956}, {3., 12.5615}}	{20.792, 54.1677}	inf	3959.	21 147.3	{4457.7, 52 443.5}	437.483	0.588184	newly	3670.	22 244.5	{4183., 43 676.}	511.592	0.629107	Reff	2.71599	0.611967	{0.156468, 1.23488}	77.468	ar	93 273.	143 003.	{93 481., 203 383.}	59.4522
31	R0	{2.46831, 8.14308}	{2.50871, 6.40674}	{{2.16856, 4.21332}, {3., 9.75362}}	{15.7491, 41.3317}	inf	1290.	18 946.6	{6020.27, 34 219.8}	1373.99	1.07549	newly	2181.	26 833.6	{5750., 72 998.}	1137.23	0.950944	Reff	2.41433	1.14641	{0.318815, 2.18406}	52.5162	ar	88 762.	162 268.	{99 882., 235 894.}	85.3032
32	R0	{1.93236, 7.38987}	{2.05253, 4.39781}	{{0.0416951, 0.892685}, {3., 5.72885}}	{56.1503, 50.3425}	inf	2899.	4625.15	{844., 7585.98}	102.872	0.324022	newly	4137.	4762.37	{748., 8478.}	84.197	0.389891	Reff	1.80908	0.262891	{0.00555485, 0.462168}	85.4682	ar	77 654.	159 599.	{103 634., 181 583.}	114.609
33	R0	{2.82251, 17.6954}	{2.42773, 6.49808}	{{1.43995, 3.30507}, {3., 10.0926}}	{20.5268, 63.2781}	inf	8313.	3118.34	{670.404, 7652.35}	81.9277	0.788071	newly	4307.	4262.93	{588., 6496.}	114.642	0.684303	Reff	2.44362	0.565017	{0.117822, 1.43821}	76.9234	ar	92 745.	187 459.	{173 137., 221 445.}	102.123
34	R0	{1.87083, 12.3994}	{2.72832, 4.63618}	{{2.16612, 1.93252}, {3., 6.17319}}	{45.8348, 62.6096}	inf	6523.	3040.21	{1996.72, 6780.81}	68.665	0.479512	newly	4361.	5221.3	{1449., 6878.}	104.238	0.355637	Reff	1.57679	0.508057	{0.164495, 0.756217}	71.5318	ar	76 282.	181 494.	{171 620., 195 576.}	137.926
35	R0	{2.95779, 16.3003}	{2.43782, 6.89353}	{{1.42269, 3.33028}, {3., 10.0698}}	{19.0925, 57.7092}	inf	6128.	1555.87	{519.943, 1597.18}	77.6903	0.7646	newly	3852.	1962.1	{705., 1871.}	84.9138	0.659605	Reff	2.67834	0.560367	{0.219317, 0.924502}	79.0778	ar	93 873.	179 102.	{171 710., 186 425.}	90.7914
36	R0	{2.18718, 14.261}	{2.6461, 11.9122}	{{2.10132, 4.71036}, {3., 17.2597}}	{29.7382, 36.8011}	inf	9128.	3460.88	{810.797, 2609.29}	84.5168	0.714744	newly	5307.	4287.26	{544., 2667.}	127.053	0.76648	Reff	1.78461	0.675702	{0.261508, 0.634102}	65.6287	ar	84 379.	182 823.	{169 496., 212 198.}	116.669
37	R0	{2.44912, 9.31118}	{2.21514, 4.23207}	{{1.01881, 2.65278}, {3., 4.94133}}	{34.6092, 54.5487}	inf	3260.	13 937.4	{4791.85, 32 310.3}	331.025	0.517096	newly	3995.	14 913.1	{4970., 39 483.}	277.131	0.427706	Reff	2.30876	0.34302	{0.0447012, 0.731757}	85.1427	ar	88 442.	161 712.	{90 342., 192 892.}	85.009
38	R0	{2.05949, 5.72514}	{2.17784, 4.03845}	{{1.04676, 2.64617}, {3., 4.96644}}	{42.3192, 30.6871}	inf	809.	15 683.6	{5109.47, 49 139.3}	1840.54	1.127	newly	2064.	16 704.3	{6583., 57 149.}	712.554	0.752643	Reff	2.02491	0.33909	{0.0554132, 0.728446}	83.2541	ar	81 587.	169 125.	{96 918., 215 254.}	108.847
39	R0	{1.75505, 8.52404}	{2.37006, 5.77078}	{{0.304904, 3.46313}, {3., 11.8109}}	{57.1733, 49.2664}	inf	4468.	8647.77	{167.07, 16 510.}	138.872	0.407728	newly	4543.	7841.96	{167., 14 078.}	133.703	0.481249	Reff	1.55847	0.565799	{0.0710231, 0.705878}	65.7735	ar	70 925.	190 424.	{169 041., 236 216.}	168.486
40	R0	{2.24724, 13.7861}	{2.58714, 12.3094}	{{2.08555, 4.85765}, {3., 18.7584}}	{25.5659, 42.9573}	inf	10 101.	4648.75	{1835.26, 3423.13}	84.3365	0.621893	newly	6145.	5881.3	{759., 2969.}	140.241	0.736934	Reff	1.78404	0.5524	{0.0560808, 2.08553}	72.7544	ar	85 257.	192 660.	{175 706., 258 130.}	125.975
41	R0	{1.77635, 8.62406}	{2.12789, 8.50814}	{{0.35038, 2.72902}, {3., 12.6246}}	{63.2013, 44.7418}	inf	5330.	4768.58	{153.424, 10 766.}	116.152	0.830858	newly	5334.	4248.34	{72., 20 038.}	119.891	0.969976	Reff	1.53452	0.458488	{0.0694541, 0.632292}	71.9143	ar	72 941.	210 832.	{195 261., 261 814.}	189.045
42	R0	{1.80397, 5.4932}	{2.2957, 4.28837}	{{1.14328, 2.64751}, {3., 4.95334}}	{51.5554, 28.7443}	inf	2276.	16 348.7	{4937.22, 46 170.9}	621.18	0.714763	newly	4341.	18 540.1	{5143., 49 207.}	332.873	0.464095	Reff	1.7106	0.378588	{0.0586601, 0.857609}	77.8681	ar	73 232.	159 720.	{66 492., 226 134.}	125.045
43	R0	{2.83096, 16.7077}	{2.2164, 7.54712}	{{0.850784, 0.707209}, {3., 18.3301}}	{27.6798, 61.618}	inf	8628.	7263.58	{1543.01, 7123.31}	47.4432	0.260731	newly	4809.	8195.28	{513., 14 517.}	120.022	0.45885	Reff	2.43862	0.413683	{0.132549, 0.599414}	83.0362	ar	92 740.	187 929.	{173 982., 229 609.}	102.641
44	R0	{2.73678, 11.6021}	{1.85738, 3.89002}	{{0.00926157, 0.903885}, {3., 5.79316}}	{41.3658, 67.0137}	inf	6552.	5143.27	{589.739, 17 132.2}	74.1549	0.471338	newly	6185.	5662.2	{515., 16 762.}	78.3334	0.496891	Reff	2.44682	0.243767	{0.000764663, 0.444346}	90.0374	ar	92 003.	158 236.	{92 958., 195 187.}	76.9343
45	R0	{2.14235, 6.98601}	{2.45458, 4.64981}	{{1.995, 1.50563}, {3., 7.28941}}	{25.9092, 37.215}	inf	2850.	2491.85	{558.85, 2814.13}	85.245	0.414427	newly	4808.	2942.83	{729., 4558.}	79.3546	0.568315	Reff	2.02463	0.522301	{0.166306, 1.1348}	74.2026	ar	82 974.	182 825.	{165 883., 218 952.}	120.34
46	R0	{2.23409, 9.21289}	{2.52497, 4.89612}	{{2.00059, 1.47696}, {3., 7.27611}}	{25.7822, 46.8557}	inf	2544.	2128.32	{882.319, 2602.9}	72.0352	0.337719	newly	3017.	3565.5	{698., 4563.}	116.221	0.465823	Reff	2.12542	0.52574	{0.166238, 1.25284}	75.2642	ar	85 163.	198 509.	{181 727., 256 301.}	133.093
47	R0	{2.12258, 8.15265}	{2.13528, 4.21067}	{{0.992475, 2.64769}, {3., 4.90996}}	{40.6053, 49.4531}	inf	2945.	15 567.	{5538.93, 54 535.3}	435.835	0.595095	newly	3839.	17 540.8	{3393., 56 393.}	364.162	0.476774	Reff	1.99728	0.349501	{0.0577392, 0.667089}	82.5012	ar	82 690.	154 439.	{90 888., 192 985.}	91.827
48	R0	{2.71771, 10.4636}	{2.13257, 4.25095}	{{1.04597, 2.64615}, {3., 4.9533}}	{31.2945, 59.3739}	inf	2061.	14 558.	{4820.57, 30 173.4}	606.851	0.710878	newly	2437.	15 983.9	{6613., 36 976.}	556.619	0.653517	Reff	2.62862	0.37821	{0.117506, 0.773143}	85.6119	ar	91 716.	152 552.	{60 272., 247 011.}	80.2376
49	R0	{2.44369, 6.66477}	{2.57956, 6.86578}	{{2.16451, 4.16848}, {3., 9.85469}}	{19.3163, 41.8287}	inf	1213.	22 871.	{4792.65, 54 138.6}	1786.55	1.13854	newly	3032.	22 386.8	{5192., 57 612.}	640.34	0.738649	Reff	2.39345	0.906875	{0.308673, 2.03082}	62.1101	ar	88 827.	137 555.	{63 007., 218 983.}	66.7615
50	R0	{2.77383, 17.9775}	{2.39827, 5.85801}	{{0.597417, 0.906033}, {3., 18.3756}}	{23.9761, 68.4713}	inf	14 883.	6762.05	{1735.72, 7197.4}	65.7814	0.582774	newly	6948.	9279.5	{592., 12 682.}	90.0725	0.505459	Reff	2.0682	0.491192	{0.106366, 0.600196}	76.5604	ar	92 334.	182 811.	{173 313., 184 754.}	97.9886
